# A surrogate measure for patient reported symptom remission in administrative data

**DOI:** 10.1186/s12888-021-03133-1

**Published:** 2021-03-04

**Authors:** Farrokh Alemi, Mai Aljuaid, Naren Durbha, Melanie Yousefi, Hua Min, Louisa G. Sylvia, Andrew A. Nierenberg

**Affiliations:** 1 Dept. of Health Administration and Policy, College of Health and Human Services, George Mason University, Fairfax, USA; 2 School of Nursing, College of Health and Human Services, George Mason University, Fairfax, USA; 3 Dauten Family Center for Bipolar Treatment Innovation, Department of Psychiatry, Massachusetts General Hospital, Boston, MA USA; 4 Harvard Medical School, Boston, MA USA

**Keywords:** Prior medication use, Medication augmentation, Medication switch, Therapeutic dose, Antidepressant effectiveness, Symptom remission, STAR*D

## Abstract

**Background:**

In real-world pragmatic administrative databases, patient reported remission is often missing.

**Objective:**

We evaluate if, in administrative data, five features of antidepressant use patterns can replace patient-reported symptom remission.

**Method:**

We re-examined data from Sequence Treatment Alternatives to Relieve Depression (STAR*D) study. Remission was measured using 50% reduction in Hamilton index. Pattern of antidepressant use was examined through five variables: (a) number of prior ineffective antidepressants, (b) duration of taking current antidepressant, (c) receiving therapeutic dose of the medication, and (d) switching to another medication, or (e) augmenting with another antidepressant. The likelihood ratio (LR) associated with each of these predictors was assessed in 90% of data (3329 cases) and evaluated in 10% of data (350 cases) set-aside for evaluation. The accuracy of predictions was calculated using Area under the Receiver Operating Curve (AROC).

**Results:**

Patients who took antidepressants for 14 weeks (LR = 2.007) were more likely to have symptom remission. Prior use of 3 antidepressants reduced the odds of remission (LR = 0.771). Patients who received antidepressants below therapeutic dose were 5 times less likely to experience remission (LR = 0.204). Antidepressant that were augment or switched, almost never led to remission (LR = 0.008, LR = 0.002 respectively). Patterns of antidepressant use accurately (AROC = 0.93) predicted symptom remission.

**Conclusion:**

Within the first 100 days, antidepressants use patterns could serve as a surrogate measure for patient-reported remission of symptoms.

## Introduction

Well-designed observational studies can complement, and in some occasions replace, clinical trials of effectiveness of antidepressants [1, 2]. Observation studies rely on large sample size to detect side-effects and risks associated with taking antidepressants [3]. These studies can show how effectiveness of antidepressants rely on patients’ multiple comorbidities [4]. Observational studies can also facilitate the examination of antidepressants effectiveness subsets of patients, clarifying differences in reaction to antidepressants among nationality, ethnic, and racial minorities. Despite the obvious advantages of studying comparative effectiveness of antidepressants through observational studies, few such studies are done; in part, because the most crucial outcome of antidepressant use, i.e. patient-reported remission of symptoms, is not typically available in Electronic Health Records.

The effort to create surrogate measure for remission of symptoms is not new. Investigators have organized complex scoring systems to capture likelihood of remission in bipolar depression [5]. Some studies use interaction between antidepressants and various other medications [6–8]. Many studies use antidepressant duration/discontinuation as a marker of effectiveness [9–15]. A number of investigators have suggested that effectiveness of antidepressants should be measured at the level of function and not symptom remission [16]. By function, it is often understood to include employment and maintenance of family unit. In claims data, social functioning is rarely coded, but if coded these variables show as Z and V codes. In this paper, we rely on five features of antidepressant-use as a predictor of remission:

- Duration of use,
- Failure to reach therapeutic dose levels,
- Augmentation of antidepressants with other medications [17, 18]
- Switch from one antidepressant to another [19]
- Treatment resistance as measured by prior use of multiple antidepressants [20].

This study reports how accurately these patterns of antidepressant use predict patient’s self-reported symptom remission.

## Methods

This project was reviewed by George Mason University IRB and was considered exempt because it relied on de-identified and publicly available data.

### Source of data

The Sequenced Treatment Alternatives to Relieve Depression (STAR*D) Study, is a public health clinical trial that is funded by the National Institute of Mental Health [21]. The STAR*D study is the largest community-based effectiveness study of antidepressant use that also reports symptom remission. Pattern of antidepressant use is available until 14 weeks after prescription and therefore, long-term use cannot be evaluated.

Details of demographic and clinical features of the STAR*D participants have been published previously in multiple papers [22]: 75.8% of participants were White, 17.6% Black, 13.0% were Hispanic, 41.7% were married, 26.5% were divorced, and 63.7% were female. The mean age was 40.8 years (standard deviation of 13.0 years). 62% of the participants were from psychiatric care settings. Depressive symptoms were moderate to severe. 75% of the patients had recurrent or chronic depression with mean length of depression of 15.5 years, with an average of 3.3 general medical comorbidities.

The STAR*D data were randomly divided into two sets: training and testing. Specifically, 90% of the data were used for training, i.e. estimating the parameters of the model; and the remaining 10% of data were used for testing the accuracy of the model.

### Timing of variables

We focus on predicting symptom remission in the first 100 days after start of the antidepressant. This constitutes a short-term evaluation of remission, during a period when patients actively try different medications.

### Calculation of likelihood ratio

The likelihood ratio (LR) calculates how many times symptom remission is more likely when a feature is present. It was calculated from the training data set using the following formula [23]:

$$ {\mathrm{LR}}_{Feature}=\frac{\mathrm{Prevalence}\ \mathrm{of}\ \mathrm{feature}\ \mathrm{for}\ \mathrm{patients}\ \mathrm{with}\ \mathrm{remission}}{\mathrm{Prevalence}\ \mathrm{of}\ \mathrm{feature}\ \mathrm{for}\ \mathrm{patients}\ \mathrm{with}\mathrm{out}\ \mathrm{remission}} $$

A ratio larger than 1 increases the odds of symptom remission. A ratio less than 1 decreases the odds of symptom remission.

### Prediction of remission

The odds of remission was calculated as the prior odds times product of the likelihood ratios associated with the patient’s features:

$$ \mathrm{Odds}\ \mathrm{of}\ \mathrm{Remission}=\mathrm{Prior}\ \mathrm{Odds}\ast {\prod}_{\begin{array}{c}\mathrm{Patien}{\mathrm{t}}^{\prime}\mathrm{s}\\ {}\mathrm{Features}\end{array}}{\mathrm{LR}}_{\mathrm{Feature}} $$

For instance, consider a patient who has taken 2 prior unsuccessful antidepressants (LR = 0.771) and then takes a third antidepressant for 14 weeks (LR = 2.44). The prior odds in this example was calculated as 0.6. Then, the odds of remission is calculated as 0.6*0.771* 2.44 = 1.129, which corresponds to a probability of experiencing remission of 0.53.

### Test of accuracy

In the 10% set aside validation data (treatment episodes of 350 cases), the accuracy of the prediction of remission, in the first 100 days, was calculated using the Area under the Receiver Operating Curve (AROC) [22]. These curves are constructed from the sensitivity and specificity of the predictions at different cutoff levels. The AROC of 1 is perfect prediction and AROC of 0.5 indicates random prediction.

## Results

**Fig. 1:**
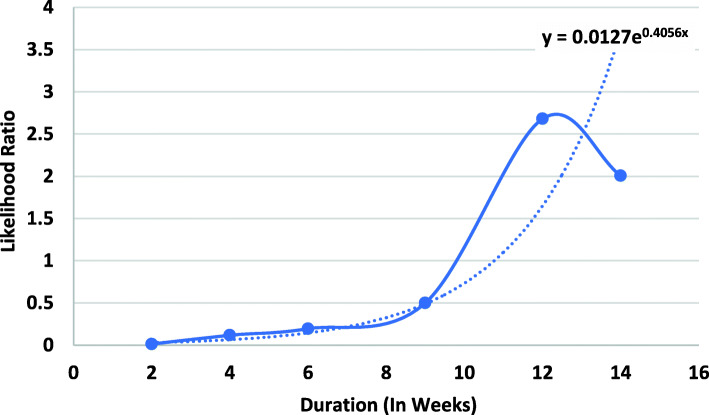
Likelihood Ratio of Remission and Duration of Medication

Figure 1 shows the likelihood ratios associated with duration of taking antidepressants. The longer the patient takes their medication, the more likely they would be to experience symptom remission. A likelihood ratio greater than 1 is observed only after 12 weeks of using the antidepressant, highlighting that remission does not tend to occur when the medication is taken for short duration. There are variations in the data (for example, after 14th weeks the likelihood ratio was lower than the value for 12 weeks). Across the entire data, the best line fitted to the data suggests that the longer one takes the antidepressant the more likely they will have remission.

Table 1 shows the LR of remission associated with different characteristics of antidepressant use, using the data in the training data set. Patients who switched to another antidepressant were unlikely to have experienced remission (LR=1/283) prior to the switch. It did not matter if patients switched or augmented their medication (LR = 0.008). In either case, few or no patients experienced remission. Patients who neither switched nor had an augmentation were nearly equally likely to experience remission (LR = 1.039). These data suggest that a switch or augmentation typically occurs after a prior medication has not been successful.

**Table 1:** Symptom Remission Given Characteristics of Antidepressant Use. (Calculated from Remission Rate in Treatment Episodes of 3329 Training Cases)

Variable	Level	Likelihood Ratio	Number with Remission	Number without Remission
Duration	Duration of 2 weeks	0.013	4	461
	Duration of 4 weeks	0.017	31	409
	Duration of 6 weeks	0.195	71	561
	Duration of 9 weeks	0.499	213	660
	Duration of 12 weeks	2.680	1030	595
	Duration of 14 weeks	2.007	770	594
Switch or Augmentation	Switch in the Same Family	1/283	0	282
	Switch to a Different Family	0.002	0	1115
	No Switch & Not Augmented	1.039	1464	2399
	No Switch & Augmented	0.008	3	601
Therapeutic Level	Met Therapeutic Level	0.982	2093	3627
	Did Not Meet Therapeutic Level	0.204	63	525
History of Antidepressant Use	1st Antidepressant	1.039	1469	2406
	2nd Antidepressant	1	518	881
	3rd Antidepressant	0.771	107	236
	4th Antidepressant	0.766	36	80
	4+ Antidepressant	0.661	26	67
	Total Episodes		2156	3670

Patients who had not taken any antidepressants previously were most likely to experience remission. As the number of prior antidepressants increased, the likelihood ratio of remission decreased (Table 1). If the patient had taken 4 antidepressants, then he/she was 1.5 times less likely to experience remission (LR = 0.667).

Table 1 also shows the impact of reaching therapeutic dose levels at any time during the course of the treatment. Patients whose antidepressant met the minimum therapeutic level were nearly equally likely to experience remission or no remission (LR = 0.982). Patients whose medication did not meet the minimum therapeutic level were nearly five times less likely to experience remission (LR = 0.204).

For ease of understanding, the probability scores can be replaced with several simplified rules. We split each factor into a binary variable and scored various combination of these variables. We worked with clinicians to identify assignment of symptom remission based on aggregate probability of remission. These rules are described in Table 2. These rules suggest that only when anti-depressants are taken for at least 10 weeks, there is no switch or augmentation, and dose is above therapeutic limit, then remission is highly likely.

**Table 2:** Rules for Classification of Symptom Remission from Features of Antidepressant Use

Rule Number	Switched or Augmented	Duration	Dose	Prior Antidepressants	Remission
1	Yes	< 10 weeks	Not Met	3+	No
2	Yes	< 10 weeks	Not Met	1–2	No
3	Yes	< 10 weeks	Met	3+	No
4	Yes	< 10 weeks	Met	1–2	No
5	Yes	> 10 weeks	Not Met	3+	No
6	Yes	> 10 weeks	Not Met	1–2	No
7	Yes	> 10 weeks	Met	3+	No
8	Yes	> 10 weeks	Met	1–2	No
9	No	< 10 weeks	Not Met	3+	No
10	No	< 10 weeks	Not Met	1–2	No
11	No	< 10 weeks	Met	3+	No
12	No	< 10 weeks	Met	1–2	No
13	No	> 10 weeks	Not Met	3+	No
14	No	> 10 weeks	Not Met	1–2	No
15	No	> 10 weeks	Met	3+	Yes
16	No	> 10 weeks	Met	1–2	Yes

**Fig. 2:**
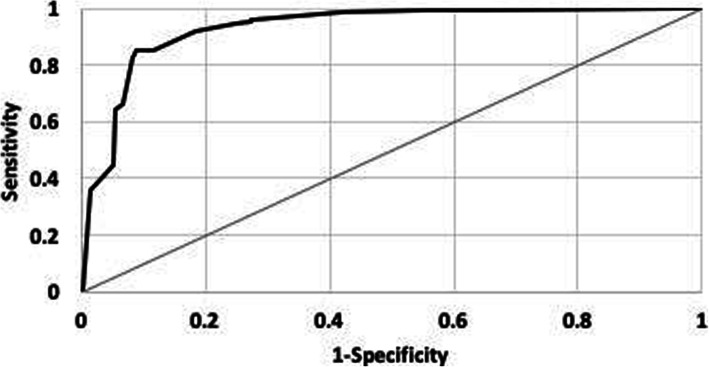
**Receiver Operating Curve for Predicting Remission.** (Predictions Were Made for Treatment Episodes of 350 Set-Aside Evaluation Cases)

The accuracy of the model was evaluated in predicting remission for treatment episodes of 350 cases, not used in estimating the likelihood ratios. The overall accuracy of predictions can be seen in Fig. 2, which shows the sensitivity and specificity of predictions at different cutoff levels. The X-axis shows one minus specificity and the Y-axis shows the sensitivity of the predictions. The further the curve is from the diagonal line, the more accurate the prediction. The AROC associated with this curve was 0.93.

### Limitations

This study has several limitations. Antidepressant use patterns may be affected by cost or administrative reasons. For example, changes in insurance formularies can affect patterns of use of antidepressants. These types of changes may confound the initiation, continuation, switching, and/or termination of antidepressants. The current study also does not address the impact of changes in formulary.

This paper did not address long-term effectiveness of antidepressants. Further research is needed to extend the proposed measure of outcome to long-term use of antidepressants. The goal of long-term use of antidepressants, i.e. use beyond first 100 days, is to prevent relapse of depression. Therefore, for long-term effectiveness of antidepressants we propose to use days till (a) new diagnosis of depression, (b) intentional self-harm/suicide, (c) premature death, (d) certain side-effects and complications of use of antidepressants, e.g. falls, sleeping problems, sexual dysfunctions, bleeding [24, 25], (e) abandoning treatment against medical advice, and (f) psychiatric hospitalization.

Finally, it may not be reasonable to assume that what worked in STAR-D study may work for other settings. In day-to-day practice, the guides to dosing given in STAR*D does not exist. Any special rules for trying to prevent study dropout does not exist. The clinicians in STAR*D may have tried harder to accomplish remission because they were aware their effort was being studied.

## Discussion

Patterns of antidepressant use nearly perfectly (AROC=0.93) predicted symptom remission. Therefore, these patterns can be used as a surrogate measure of remission. Specifically, patients taking medications for less than 12 weeks, who take antidepressants below the therapeutic level, who have had more than 2 prior antidepressants that were not successful, who switched or augmented their antidepressants are less likely to experience symptom remission.

Given the level of experimentation that goes on within the first 100 days of use of antidepressants, it is not surprising that patterns of use predict remission. The study agrees with widely held intuition that short use of antidepressants is a clear indication of lack of benefit. Why continue with a medication, if it is not working?

This study also provides support for the intuition that when a medication is continued, sometimes there is remission and other times there is not. Some patients may continue with their medications, even when they do not experience remission of their symptoms. Other patients experience remission. By 14th week, if patients continue to use their antidepressant, they are twice more likely (LR = 2.007) to have experienced remission. While continuation does not guarantee remission, it increases the odds of remission.

This paper has shown that antidepressant use patterns are an accurate, although probabilistic, predictor of remission during the first 100 days of a depression episode. Future research can compare different methods of evaluating antidepressant outcomes. Of particular interest would be to contrast (1) patient reported remission, (2) remission as predicted from antidepressant use patterns, (3) patient’s ability to function at home/work, and (4) other outcomes of use of antidepressants.

## Data Availability

Data are publicly available from National Institute of Mental Health Data Archive at https://nda.nih.gov/edit_collection.html?id=2148.

## Abbreviations

AROC: Area under the Receiver Operating Curve
LR: Likelihood Ratio
STAR*D: Sequence Treatment Alternatives to Relieve Depression study
